# Crystal structure and thermal properties of bis­[μ-2-(meth­oxy­carbonyl­hydrazinyl­idene)acetato-κ^3^ *N* ^1^,*O*:*O*]bis­[di­aqua­(thio­cyanato-κ*N*)manganese(II)] tetra­hydrate

**DOI:** 10.1107/S2056989018014871

**Published:** 2018-10-26

**Authors:** Subbiyan Poornima, Thathan Premkumar, Raymond J. Butcher, Subbaiah Govindarajan

**Affiliations:** a Department of Chemistry, Bharathiar University, Coimbatore 641 046, Tamilnadu, India; b The University College, Sungkyunkwan University, Suwon 440-746, Republic of Korea; c Department of Chemistry, Sungkyunkwan University, Suwon 440-746, Republic of Korea; d Department of Chemistry, Howard University, Washington, DC 20059, USA

**Keywords:** Schiff base, manganese(II) complex, thermal decomposition, crystal structure

## Abstract

The title compound, [Mn_2_(C_4_H_5_N_2_O_4_)_2_(NCS)_2_(H_2_O)_4_]·4H_2_O (I), exists as a centrosymmetric dimer. Each dimeric unit consists of tridentate (*O*,*O*,*N*)-chelating Schiff bases with symmetry-maintained μ-*O*-bridged carboxyl­ate anions, terminally bound thio­cyanate anions, and ligated and solvated water mol­ecules. The complex exhibits a distorted octa­hedron geometry and the centrosymmetric μ-*O*-bridged carboxyl­ate anions connect the two manganese atoms to form an *M*_2_O_2_ ring. In the crystal, the mol­ecules are inter­linked *via* strong N—H⋯O and O—H⋯O hydrogen-bonding contacts and weak O—H⋯S inter­molecular inter­actions, forming a three-dimensional mol­ecular network.

## Abstract 2

The title compound was prepared by a template method starting from manganese(II) nitrate with a Schiff base ligand. The product of condensation was between methyl carbazate and glyoxylic acid, and formed *in situ* in aqueous solution containing ammonium thio­cyanate. The manganese compound crystallized in the monoclinic space group *P2_1_/n* and exists as a centrosymmetric dimer.

## Chemical context   

Hydrazine, di­nitro­gen tetra­hydride (N_2_H_4_), is the simplest di­amine and parent of innumerable organic derivatives. Among them, carbaza­tes (esters of hydrazine­carb­oxy­lic acid, NH_2_-NH-COO-*R*, where *R* = CH_3_, C_2_H_5_, CH_2_C_6_H_5_ *etc*) are inter­esting as ligands in view of their variety of potential donor atoms such as oxygen and nitro­gen. Inter­estingly, these neutral mol­ecules can be expected to exhibit only one common coordination mode, *i.e. N*,*O*-chelating bidentate. This has been clearly observed in many metal complexes with a variety of anions such as formate (Srinivasan *et al.*, 2011 ▸), benzoate (Kathiresan *et al.*, 2012 ▸), thio­cyanate (Srinivasan *et al.*, 2014*a* ▸,*b* ▸), nitrate (Zhang *et al.*, 2005 ▸; Srinivasan *et al.*, 2007 ▸,2008 ▸) and perchlorate (Chen *et al.*, 2016 ▸, Sitong *et al.*, 2016 ▸). Apart from their coordination ability, alkyl carbaza­tes can also undergo condensation reactions; the hydrazinic part of the terminal amine group can react with the carbonyl group of aldehydes or ketones to form Schiff bases. In this regard, Schiff bases and their Co^III^, Ni^II^, Pd^II^ and Fe^II^ complexes based on (2-phenyl­phosphino)benzaldehyde with ethyl carbazate (Milenković *et al.*, 2013*a* ▸,*b* ▸, 2014 ▸) have been reported. Recently, we have also reported Schiff bases generated from analogous benzyl carbazate with alkyl and heteroaryl ketones, and their metal complexes (Nithya *et al.*, 2016 ▸, 2017*a* ▸,*b* ▸, 2018*a* ▸,*b* ▸). However, no work involving Schiff base complexes of alkyl carbaza­tes with an aldehydic, or α-keto acid, has been reported so far, except from our own recent report of a Schiff base generated from methyl carbazate and α-ketoglutaric acid, and its silver(I) complex (Parveen *et al.*, 2018 ▸). In a continuation of our investigations, the title complex (I) was prepared by a template method starting from manganese(II) nitrate with a Schiff base ligand. The product of condensation between methyl carbazate and glyoxylic acid, formed *in situ* in aqueous solution containing ammonium thio­cyanate.
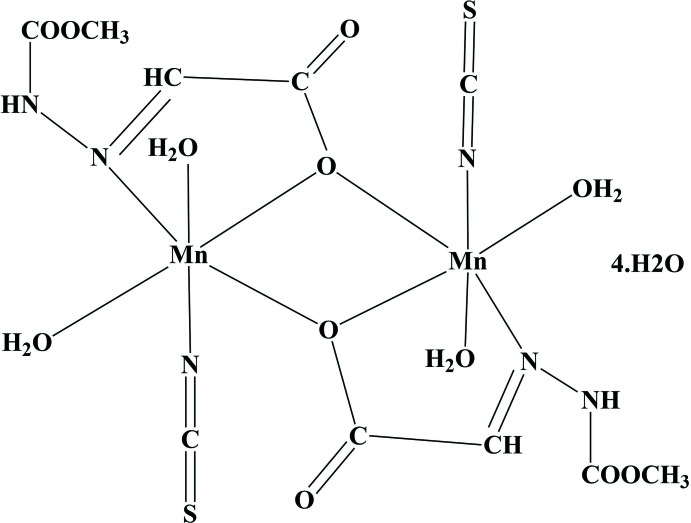


## Structural commentary   

### General structural details   

**Figure 1:**
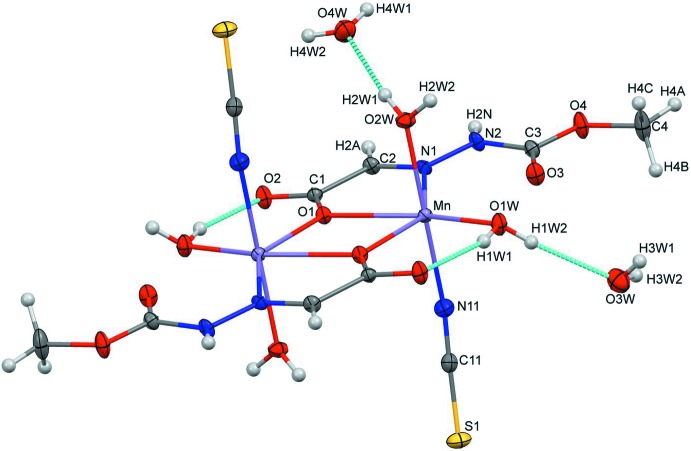
Mol­ecular structure of the title complex (I), showing the atom-numbering scheme and displacement ellipsoids drawn at the 50% probability level. The mol­ecule is located about an inversion centre and the unlabelled atoms are generated by the symmetry operation (−*x* + 1, −*y*, −*z* + 1).

The manganese title compound crystallizes in the monoclinic space group *P2_1_/n* and exists as a centrosymmetric dimer (Fig. 1 ▸). The asymmetric unit consists of an Mn atom, a tridentate Schiff base ligand, an N-bounded thio­cyanate moiety, and two ligated and two solvated water mol­ecules. The manganese atom is surrounded in a distorted octa­hedral geometry by symmetry-related μ-*O-*bridged carboxyl­ate anions, one azomethine nitro­gen, an N-bounded NCS anion and two ligated water mol­ecules with an MnN_2_O_4_ core. The axial sites are occupied by one of the coordinated water mol­ecules (O2*W*) and the N-bonded NCS anion, whereas the μ-*O-*bridged carboxyl­ate anions, azomethine nitro­gen atom and a coordinated water mol­ecule (O1*W*) occupy the equatorial positions. The two manganese atoms are connected *via* centrosymmetrically related μ-*O*-bridged carboxyl­ate anions, forming a rhomboidal Mn_2_O_2_ unit about an inversion centre.

### Specific structural details   

The separation of the Mn atoms is 3.645 (3) Å. The Mn—N(iso­thio­cyanato) and Mn—N(azomethine) distances are 2.1289 (11) and 2.3388 (10) Å and the Mn—O distances involving the coordinated water mol­ecules and μ-*O*-bridged carboxyl­ate anions are 2.1448 (9), 2.1905 (9) and 2.2606 (8), 2.2985 (8) Å, respectively. The Mn—N—C—S torsion angle in the NCS moiety is 103.5 (4)° and the bond angles for the coordinated atoms vary from 68.99 (3)–132.57 (4)°, indicating a distorted geometry.

## Supra­molecular features   

**Figure 2:**
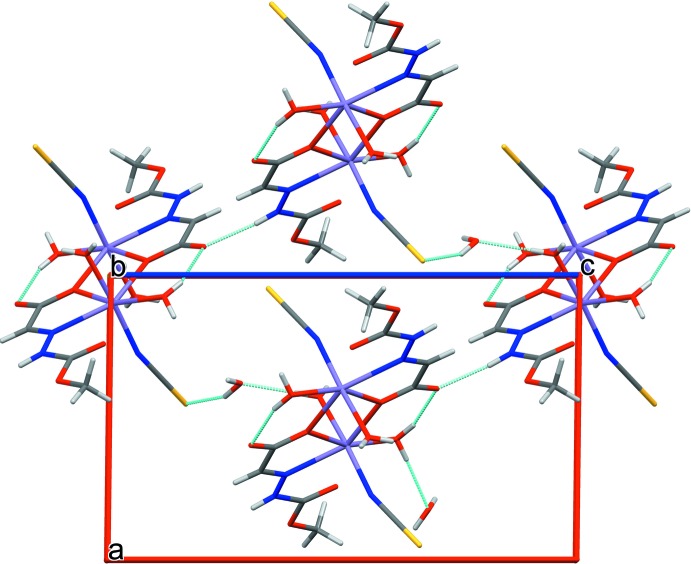
View of a two-dimensional array of (I) showing N—H⋯O and O—H⋯O hydrogen bonds and weak O—H⋯S inter­molecular inter­actions (green lines) in a projection along the *b* axis.

**Figure 3:**
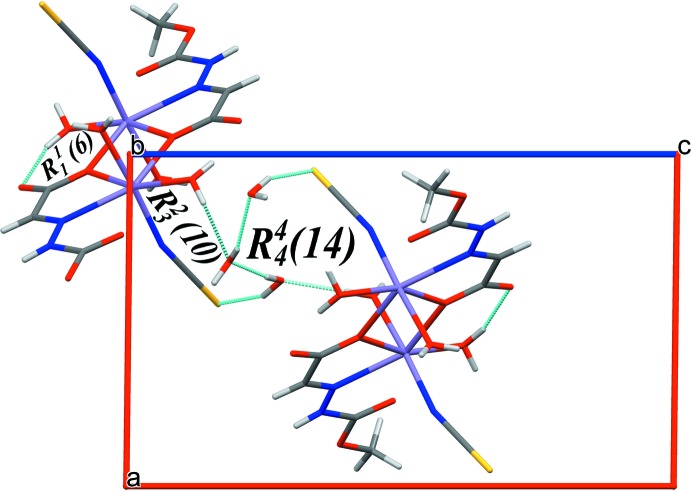
View of hydrogen-bonding inter­actions (green lines) along the *ac* plane forming various ring motifs to further stabilize the three-dimensional network.

**Figure 4:**
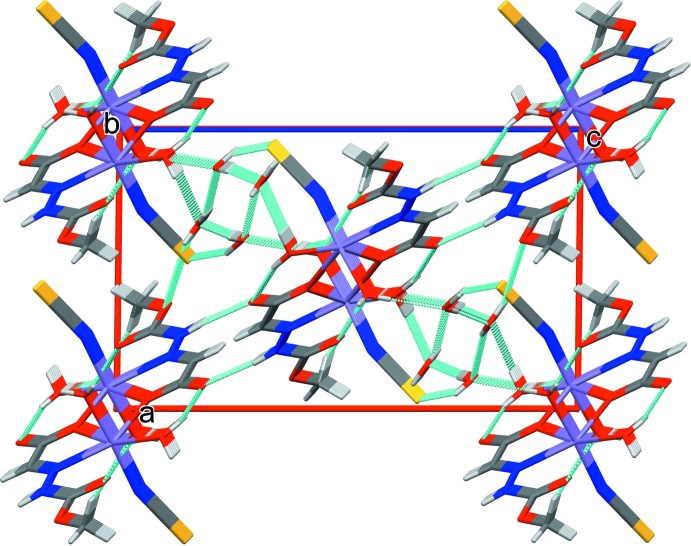
Overall packing view of the three-dimensional network for (I), viewed along the *b* axis, showing N—H⋯O and O—H⋯O hydrogen bonds and weak O—H⋯S inter­molecular inter­actions (green lines) and the stacking of (I) along the *b* axis.

The crystal structure of (I) contains both coordinated and solvated water mol­ecules. Inter- and intra-mol­ecular hydrogen-bonding inter­actions (Table 1 ▸) stabilize the supra­molecular three-dimensional network. The N2—H2*N*⋯O2^v^ [2.7971 (13) Å] hydrogen bond between adjacent dimers forms chains extending along the *ac* diagonal. The weak O4*W*—H4*W*1⋯S1^iv^ inter­action [3.3159 (12) Å] and O2*W*—H2*W*1⋯O4*W* hydrogen bond [2.7322 (14) Å] link the dimers, generating a two-dimensional network as shown in Fig. 2 ▸. The ligated and solvated water mol­ecules O1*W*, O3*W* and O4*W* are involved in O—H⋯O hydrogen-bonding inter­actions [2.733 (14)–3.2158 (15) Å, Table 1 ▸] that stack the complex mol­ecules along the *b*-axis direction. These contacts combine to generate several ring motifs (Fig. 3 ▸) *viz*. *R* ^1^_1_(6), *R* ^2^_3_(10) and *R* ^4^_4_(14) that stabilize the three-dimensional supra­molecular network (Fig. 4 ▸).

## Thermal properties   

**Figure 5:**
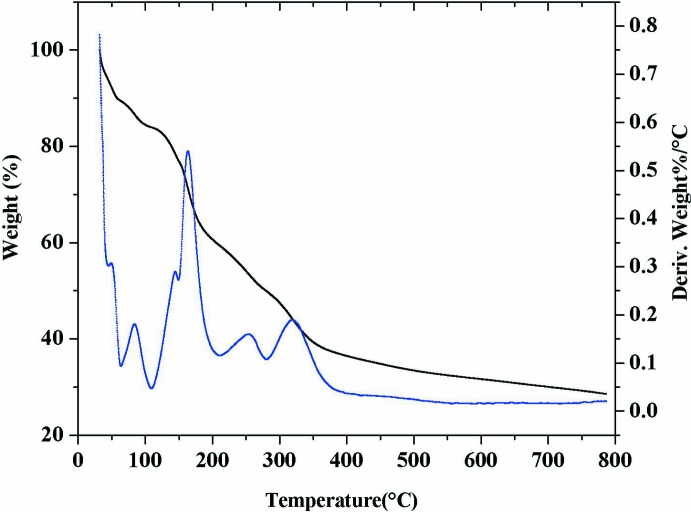
Simultaneous TG/DTG (N_2_ atmosphere) analysis of the title complex (I).

The thermal decomposition behaviour of the title complex was studied by simultaneous TG–DTG analyses recorded in a nitro­gen atmosphere in the temperature range 30–800°C, as shown in Fig. 5 ▸. The TG curve displays the combined mass loss of 20.5% (calculated 21.8%) in the temperature range 30–140°C corresponding to dehydration of both the solvated and coordinated water mol­ecules. The anhydrous compound then shows continuous decomposition between 140 and 600°C to give manganese sulfide as the end product (mass loss observed 73.6%, calculated 72.50%). The DTG curve shows a doublet (40 and 80°C) for dehydration and a multiplet (150, 164, 255 and 321°C) for the decomposition of the anhydrous compound in accordance with TG mass loss.

## Database survey   

There are a few structures of metal complexes in the crystallographic literature with simple hydrazones based on glyoxylic acid and salicyloyl hydrazine (Liu *et al.*, 2010 ▸) and thio­semicarbazide (Dodoff *et al.*, 2006 ▸; Huseynova *et al.*, 2018 ▸). In the former salicyloyl hydrazone complex of cadmium, the Schiff base acts as a tetra­dentate (*O*,*N*,*O*,*O*) ligand with one of the carboxyl­ate oxygen atoms bridging the cadmium centers, leading to a dimer, whereas in the thio­semicarbazone complexes of Zn, Pd, Pt, Co, and Ni (Milenković *et al.*, 2013*a* ▸,*b* ▸, 2014 ▸), the ligand adopts a tridentate (*O*,*N*,*O*) coordination mode. Recently, we have reported a silver(I) complex of 2-(meth­oxy­carbonyl­hydrazono)penta­nedioic acid in which the neutral as well as monoanionic Schiff base behaves as a tridentate (*O*,*N*,*O*) group, leading to an octa­hedral coordination of the silver atom (Parveen *et al.*, 2018 ▸).

## Synthesis and crystallization   

Elemental analyses for carbon, hydrogen and nitro­gen were recorded using a Vario-ELIII elemental analyzer. The IR spectrum was recorded using a JASCO-4100 spectrophotometer and KBr pellets in the range of 4000–400.00 cm^−1^. Simultaneous TG/DTG (TG/DTG) analyses were carried out using a TA instrument, SDT Q600 thermal analyzer, in a flowing nitro­gen atmosphere with a heating rate of 10°C min^−1^.

Stoichiometric qu­anti­ties of glyoxylic acid (0.184 g, 2 mmol), ethyl­carbazate (0.208 g, 2 mmol) and ammonium thio­cyanate (0.152 g, 2 mmol) were dissolved in 30 mL of double-distilled water. To this homogeneous solution, Mn(NO_3_)_2_·6H_2_O (0.287 g, 1 mmol) dissolved in 10.00 mL of double-distilled water was added dropwise, the pH of the resulting solution was noted as 3.45. The above clear solution was kept over a water-bath until the solution was reduced to *ca* 15 mL and allowed to stand at room temperature for slow crystallization. After two days, colourless rod-shaped crystals were obtained and filtered off, washed with ice-cold water and air dried. The product is soluble in water, methanol and ethanol and insoluble in diethyl ether. In the absence of ammonium thio­cyanate, the reaction did not yield any desired product. Yield: 64%. Analysis calculated for C_10_H_26_Mn_2_N_6_O_16_S_2_ (I): C, 29.25, H, 3.44, N, 13.57; found: C, 29.20; H, 3.49; N, 13.55. Metal (%): calculated 14.27 (found: 14.06), FT–IR (KBr, cm^−1^): 3520 (*b*) [ν(O—H)], 3206 (*b*) [ν(N—H)], 2096 (*s*) [ν(C≡N)], 1705 (*s*) [ν(C=O], 1627 (*m*) [ν_asym_ (C=O)], 1555 (*s*) [ν(C=N)], 1397 (*s*) [ν_sym_(C=O)], 1067 (*s*) [ν(N—N)].

## Refinement   

Crystal data, data collection and structure refinement details are summarized in Table 2 ▸. H atoms attached to carbon atoms were positioned geometrically and constrained to ride on their parent atoms, with carbon–hydrogen bond lengths of 0.95 Å for alkene C—H and 0.98 Å for CH_3_ groups, respectively. Methyl H atoms were allowed to rotate but not to tip to best fit the experimental electron density. *U*_iso_(H) values were set to a multiple of *U*_eq_(C) with 1.5 for CH_3_ and 1.2 for C—H groups, respectively. Positions and *U*_iso_ values of water and amine H atoms were freely refined.

## Supplementary Material

Crystal structure: contains datablock(s) I. DOI: 10.1107/S2056989018014871/jj2203sup1.cif

Structure factors: contains datablock(s) I. DOI: 10.1107/S2056989018014871/jj2203Isup2.hkl

CCDC reference: 1870123

Additional supporting information:  crystallographic information; 3D view; checkCIF report

## Figures and Tables

**Table 1:** Hydrogen-bond geometry (Å, °)

*D*—H⋯*A*	*D*—H	H⋯*A*	*D*⋯*A*	*D*—H⋯*A*
O1*W*—H1*W*1⋯O2^i^	0.83 (2)	1.92 (2)	2.7158 (13)	160 (2)
O1*W*—H1*W*2⋯O3*W*	0.80 (2)	2.07 (2)	2.8627 (15)	174 (2)
O2*W*—H2*W*1⋯O4*W*	0.79 (2)	1.94 (2)	2.7332 (14)	175 (2)
O2*W*—H2*W*2⋯O3^ii^	0.82 (3)	2.11 (3)	2.8852 (13)	159 (2)
O2*W*—H2*W*2⋯O1*W* ^ii^	0.82 (3)	2.54 (2)	3.0865 (13)	125 (2)
O3*W*—H3*W*1⋯O4*W* ^ii^	0.79 (2)	2.09 (3)	2.8264 (17)	155 (2)
O3*W*—H3*W*2⋯O4^iii^	0.80 (3)	2.54 (3)	3.2158 (15)	144 (2)
O4*W*—H4*W*1⋯S1^iv^	0.87 (3)	2.52 (3)	3.3159 (12)	153 (2)
O4*W*—H4*W*2⋯O3*W* ^iv^	0.81 (2)	1.97 (2)	2.7754 (16)	173 (2)
N2—H2*N*⋯O2^v^	0.83 (2)	1.97 (2)	2.7971 (13)	174 (2)

Symmetry codes: (i) 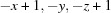; (ii) 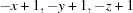; (iii) 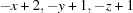; (iv) 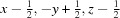; (v) 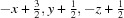.

**Table 2:** Experimental details

Crystal data	
Chemical formula	[Mn_2_(C_4_H_5_N_2_O_4_)_2_(NCS)_2_(H_2_O)_4_]·4H_2_O
*M*_r_	660.37
Crystal system, space group	Monoclinic, *P*2_1_/*n*
Temperature (K)	120
*a*, *b*, *c* (Å)	9.7060 (3), 8.3654 (3), 16.0082 (6)
β (°)	90.653 (2)
*V* (Å^3^)	1299.69 (8)
*Z*	2
Radiation type	Mo *K*α
μ (mm^−1^)	1.21
Crystal size (mm)	0.46 × 0.24 × 0.17

Data collection	
Diffractometer	Bruker APEXII CCD
Absorption correction	Multi-scan (*SADABS*; Sheldrick, 1996 ▸)
*T*_min_, *T*_max_	0.658, 0.746
No. of measured, independent and observed [*I* > 2σ(*I*)] reflections	12223, 3915, 3408
*R*_int_	0.024
(sin θ/λ)_max_ (Å^−1^)	0.715

Refinement	
*R*[*F* ^2^ > 2σ(*F* ^2^)], *wR*(*F* ^2^), *S*	0.025, 0.060, 1.05
No. of reflections	3915
No. of parameters	200
H-atom treatment	H atoms treated by a mixture of independent and constrained refinement
Δρ_max_, Δρ_min_ (e Å^−3^)	0.49, −0.34

Computer programs: *APEX2* and *SAINT* (Bruker, 2014 ▸), *SIR92* (Altomare *et al.*, 1993 ▸), *SHELXL2018/3* (Sheldrick, 2015 ▸) and *shelXle* (Hübschle *et al.*, 2011 ▸).

## supplementary crystallographic information

### Crystal data

**Table:**

[Mn_2_(C_4_H_5_N_2_O_4_)_2_(NCS)_2_(H_2_O)_4_]·4H_2_O	*F*(000) = 676
*M**_r_* = 660.37	*D*_x_ = 1.687 Mg m^−^^3^
Monoclinic, *P*2_1_/*n*	Mo *K*α radiation, λ = 0.71073 Å
*a* = 9.7060 (3) Å	Cell parameters from 6980 reflections
*b* = 8.3654 (3) Å	θ = 2.5–30.6°
*c* = 16.0082 (6) Å	µ = 1.21 mm^−^^1^
β = 90.653 (2)°	*T* = 120 K
*V* = 1299.69 (8) Å^3^	Rod, colourless
*Z* = 2	0.46 × 0.24 × 0.17 mm

### Data collection

**Table:**

Bruker APEXII CCD diffractometer	3915 independent reflections
Radiation source: fine focus sealed tube	3408 reflections with *I* > 2σ(*I*)
Graphite monochromator	*R*_int_ = 0.024
ω and phi scans	θ_max_ = 30.6°, θ_min_ = 2.4°
Absorption correction: multi-scan (SADABS; Sheldrick, 1996)	*h* = −13→12
*T*_min_ = 0.658, *T*_max_ = 0.746	*k* = −11→11
12223 measured reflections	*l* = −22→22

### Refinement

**Table:**

Refinement on *F*^2^	Primary atom site location: structure-invariant direct methods
Least-squares matrix: full	Secondary atom site location: difference Fourier map
*R*[*F*^2^ > 2σ(*F*^2^)] = 0.025	Hydrogen site location: mixed
*wR*(*F*^2^) = 0.060	H atoms treated by a mixture of independent and constrained refinement
*S* = 1.05	*w* = 1/[σ^2^(*F*_o_^2^) + (0.0256*P*)^2^ + 0.5439*P*] where *P* = (*F*_o_^2^ + 2*F*_c_^2^)/3
3915 reflections	(Δ/σ)_max_ = 0.003
200 parameters	Δρ_max_ = 0.49 e Å^−^^3^
0 restraints	Δρ_min_ = −0.34 e Å^−^^3^

### Special details

**Table:**

Geometry. All esds (except the esd in the dihedral angle between two l.s. planes) are estimated using the full covariance matrix. The cell esds are taken into account individually in the estimation of esds in distances, angles and torsion angles; correlations between esds in cell parameters are only used when they are defined by crystal symmetry. An approximate (isotropic) treatment of cell esds is used for estimating esds involving l.s. planes.

### Fractional atomic coordinates and isotropic or equivalent isotropic displacement parameters (Å^2^)

**Table:**

	*x*	*y*	*z*	*U*_iso_*/*U*_eq_	
Mn1	0.59833 (2)	0.18522 (2)	0.50685 (2)	0.00920 (5)	
S1	0.95128 (4)	−0.10626 (4)	0.66382 (2)	0.02029 (8)	
O1	0.55108 (9)	−0.03030 (10)	0.42219 (5)	0.01108 (16)	
O2	0.60023 (10)	−0.15606 (10)	0.30205 (5)	0.01546 (18)	
O3	0.74296 (10)	0.44631 (11)	0.48516 (5)	0.01696 (18)	
O4	0.87386 (10)	0.57439 (11)	0.38944 (6)	0.0191 (2)	
O1W	0.57632 (11)	0.34713 (11)	0.61341 (6)	0.01577 (18)	
H1W1	0.528 (2)	0.303 (3)	0.6488 (14)	0.046 (6)*	
H1W2	0.646 (2)	0.379 (3)	0.6349 (14)	0.041 (6)*	
O2W	0.42696 (10)	0.30284 (11)	0.44755 (6)	0.01524 (18)	
H2W1	0.414 (2)	0.292 (2)	0.3989 (13)	0.028 (5)*	
H2W2	0.396 (2)	0.389 (3)	0.4635 (15)	0.055 (7)*	
O3W	0.82278 (12)	0.48163 (13)	0.68375 (7)	0.0247 (2)	
H3W1	0.787 (3)	0.562 (3)	0.6975 (15)	0.053 (7)*	
H3W2	0.887 (3)	0.509 (3)	0.6565 (16)	0.062 (8)*	
O4W	0.38041 (13)	0.28790 (14)	0.27902 (7)	0.0265 (2)	
H4W1	0.426 (3)	0.353 (3)	0.2471 (15)	0.057 (7)*	
H4W2	0.370 (2)	0.206 (3)	0.2527 (14)	0.044 (6)*	
N1	0.70220 (10)	0.21438 (11)	0.37662 (6)	0.01068 (19)	
N2	0.77597 (11)	0.34553 (12)	0.35488 (6)	0.0123 (2)	
H2N	0.8177 (18)	0.349 (2)	0.3097 (11)	0.018 (4)*	
N11	0.77393 (12)	0.06901 (13)	0.55969 (7)	0.0184 (2)	
C11	0.84668 (13)	−0.00547 (15)	0.60238 (8)	0.0140 (2)	
C1	0.60689 (12)	−0.03982 (14)	0.35114 (7)	0.0107 (2)	
C2	0.68949 (12)	0.10135 (14)	0.32384 (7)	0.0117 (2)	
H2A	0.730139	0.106396	0.270198	0.014*	
C3	0.79375 (13)	0.45612 (14)	0.41613 (7)	0.0125 (2)	
C4	0.89246 (17)	0.70729 (17)	0.44739 (9)	0.0256 (3)	
H4A	0.947018	0.791232	0.420758	0.038*	
H4B	0.940626	0.669641	0.497796	0.038*	
H4C	0.802229	0.750334	0.462554	0.038*	

### Atomic displacement parameters (Å^2^)

**Table:**

	*U*^11^	*U*^22^	*U*^33^	*U*^12^	*U*^13^	*U*^23^
Mn1	0.01079 (9)	0.00955 (9)	0.00729 (8)	0.00015 (6)	0.00089 (6)	−0.00039 (6)
S1	0.01857 (16)	0.02354 (17)	0.01866 (15)	0.00707 (12)	−0.00337 (12)	0.00329 (13)
O1	0.0138 (4)	0.0112 (4)	0.0083 (4)	−0.0019 (3)	0.0033 (3)	−0.0009 (3)
O2	0.0215 (5)	0.0138 (4)	0.0113 (4)	−0.0043 (3)	0.0066 (3)	−0.0045 (3)
O3	0.0233 (5)	0.0163 (4)	0.0114 (4)	−0.0045 (4)	0.0061 (3)	−0.0022 (3)
O4	0.0254 (5)	0.0149 (4)	0.0170 (4)	−0.0098 (4)	0.0085 (4)	−0.0039 (3)
O1W	0.0211 (5)	0.0149 (4)	0.0114 (4)	−0.0064 (4)	0.0040 (4)	−0.0029 (3)
O2W	0.0185 (5)	0.0151 (4)	0.0121 (4)	0.0051 (3)	−0.0019 (3)	−0.0013 (3)
O3W	0.0230 (6)	0.0228 (5)	0.0284 (5)	−0.0034 (4)	0.0049 (4)	0.0034 (4)
O4W	0.0377 (6)	0.0246 (5)	0.0171 (5)	−0.0057 (5)	−0.0050 (4)	0.0004 (4)
N1	0.0108 (5)	0.0092 (4)	0.0121 (4)	−0.0004 (3)	0.0019 (3)	0.0019 (4)
N2	0.0165 (5)	0.0102 (4)	0.0102 (4)	−0.0032 (4)	0.0058 (4)	0.0003 (4)
N11	0.0173 (6)	0.0175 (5)	0.0202 (5)	0.0004 (4)	−0.0033 (4)	0.0005 (4)
C11	0.0130 (6)	0.0140 (5)	0.0151 (5)	−0.0015 (4)	0.0009 (4)	−0.0018 (4)
C1	0.0115 (5)	0.0107 (5)	0.0098 (5)	−0.0003 (4)	0.0005 (4)	0.0002 (4)
C2	0.0139 (6)	0.0123 (5)	0.0090 (5)	−0.0004 (4)	0.0033 (4)	0.0002 (4)
C3	0.0125 (6)	0.0113 (5)	0.0136 (5)	−0.0009 (4)	0.0027 (4)	0.0004 (4)
C4	0.0331 (8)	0.0186 (6)	0.0254 (7)	−0.0131 (6)	0.0074 (6)	−0.0091 (5)

### Geometric parameters (Å, º)

**Table:**

Mn1—N11	2.1289 (11)	O2W—H2W2	0.82 (3)
Mn1—O2W	2.1448 (9)	O3W—H3W1	0.79 (2)
Mn1—O1W	2.1905 (9)	O3W—H3W2	0.80 (3)
Mn1—O1^i^	2.2606 (8)	O4W—H4W1	0.87 (3)
Mn1—O1	2.2985 (8)	O4W—H4W2	0.81 (2)
Mn1—N1	2.3388 (10)	N1—C2	1.2731 (15)
Mn1—O3	2.6216 (9)	N1—N2	1.3576 (14)
S1—C11	1.6390 (13)	N2—C3	1.3577 (15)
O1—C1	1.2679 (13)	N2—H2N	0.833 (17)
O2—C1	1.2515 (14)	N11—C11	1.1588 (17)
O3—C3	1.2177 (14)	C1—C2	1.4955 (16)
O4—C3	1.3318 (14)	C2—H2A	0.9500
O4—C4	1.4578 (16)	C4—H4A	0.9800
O1W—H1W1	0.83 (2)	C4—H4B	0.9800
O1W—H1W2	0.80 (2)	C4—H4C	0.9800
O2W—H2W1	0.79 (2)		
			
N11—Mn1—O2W	177.02 (4)	Mn1—O2W—H2W1	119.7 (14)
N11—Mn1—O1W	93.31 (4)	Mn1—O2W—H2W2	123.1 (17)
O2W—Mn1—O1W	88.79 (4)	H2W1—O2W—H2W2	111 (2)
N11—Mn1—O1^i^	93.09 (4)	H3W1—O3W—H3W2	105 (2)
O2W—Mn1—O1^i^	89.24 (3)	H4W1—O4W—H4W2	106 (2)
O1W—Mn1—O1^i^	83.93 (3)	C2—N1—N2	118.52 (10)
N11—Mn1—O1	91.69 (4)	C2—N1—Mn1	118.34 (8)
O2W—Mn1—O1	87.15 (3)	N2—N1—Mn1	123.15 (7)
O1W—Mn1—O1	157.45 (3)	N1—N2—C3	115.37 (10)
O1^i^—Mn1—O1	73.84 (3)	N1—N2—H2N	120.8 (12)
N11—Mn1—N1	92.90 (4)	C3—N2—H2N	123.0 (12)
O2W—Mn1—N1	84.12 (4)	C11—N11—Mn1	163.64 (10)
O1W—Mn1—N1	132.57 (4)	N11—C11—S1	178.43 (12)
O1^i^—Mn1—N1	142.48 (3)	O2—C1—O1	126.34 (11)
O1—Mn1—N1	68.99 (3)	O2—C1—C2	117.00 (10)
N11—Mn1—O3	90.32 (4)	O1—C1—C2	116.65 (10)
O2W—Mn1—O3	88.45 (3)	N1—C2—C1	116.15 (10)
O1W—Mn1—O3	69.22 (3)	N1—C2—H2A	121.9
O1^i^—Mn1—O3	153.09 (3)	C1—C2—H2A	121.9
O1—Mn1—O3	132.76 (3)	O3—C3—O4	125.80 (11)
N1—Mn1—O3	63.78 (3)	O3—C3—N2	124.05 (11)
C1—O1—Mn1^i^	134.25 (7)	O4—C3—N2	110.14 (10)
C1—O1—Mn1	119.59 (7)	O4—C4—H4A	109.5
Mn1^i^—O1—Mn1	106.16 (3)	O4—C4—H4B	109.5
C3—O3—Mn1	113.53 (8)	H4A—C4—H4B	109.5
C3—O4—C4	115.53 (10)	O4—C4—H4C	109.5
Mn1—O1W—H1W1	108.5 (15)	H4A—C4—H4C	109.5
Mn1—O1W—H1W2	116.8 (16)	H4B—C4—H4C	109.5
H1W1—O1W—H1W2	110 (2)		
			
C2—N1—N2—C3	−175.42 (11)	O2—C1—C2—N1	−175.83 (11)
Mn1—N1—N2—C3	4.17 (14)	O1—C1—C2—N1	3.73 (16)
Mn1^i^—O1—C1—O2	−7.27 (19)	Mn1—O3—C3—O4	−178.58 (10)
Mn1—O1—C1—O2	173.28 (10)	Mn1—O3—C3—N2	1.36 (16)
Mn1^i^—O1—C1—C2	173.21 (8)	C4—O4—C3—O3	−4.53 (19)
Mn1—O1—C1—C2	−6.24 (13)	C4—O4—C3—N2	175.52 (12)
N2—N1—C2—C1	−179.86 (10)	N1—N2—C3—O3	−3.54 (18)
Mn1—N1—C2—C1	0.53 (14)	N1—N2—C3—O4	176.40 (10)

Symmetry code: (i) −*x*+1, −*y*, −*z*+1.

### Hydrogen-bond geometry (Å, º)

**Table:**

*D*—H···*A*	*D*—H	H···*A*	*D*···*A*	*D*—H···*A*
O1*W*—H1*W*1···O2^i^	0.83 (2)	1.92 (2)	2.7158 (13)	160 (2)
O1*W*—H1*W*2···O3*W*	0.80 (2)	2.07 (2)	2.8627 (15)	174 (2)
O2*W*—H2*W*1···O4*W*	0.79 (2)	1.94 (2)	2.7332 (14)	174.6 (19)
O2*W*—H2*W*2···O3^ii^	0.82 (3)	2.11 (3)	2.8852 (13)	159 (2)
O2*W*—H2*W*2···O1*W*^ii^	0.82 (3)	2.54 (2)	3.0865 (13)	125 (2)
O3*W*—H3*W*1···O4*W*^ii^	0.79 (2)	2.09 (3)	2.8264 (17)	155 (2)
O3*W*—H3*W*2···O4^iii^	0.80 (3)	2.54 (3)	3.2158 (15)	144 (2)
O4*W*—H4*W*1···S1^iv^	0.87 (3)	2.52 (3)	3.3159 (12)	153 (2)
O4*W*—H4*W*2···O3*W*^iv^	0.81 (2)	1.97 (2)	2.7754 (16)	173 (2)
N2—H2*N*···O2^v^	0.833 (17)	1.967 (17)	2.7971 (13)	173.9 (17)

Symmetry codes: (i) −*x*+1, −*y*, −*z*+1; (ii) −*x*+1, −*y*+1, −*z*+1; (iii) −*x*+2, −*y*+1, −*z*+1; (iv) *x*−1/2, −*y*+1/2, *z*−1/2; (v) −*x*+3/2, *y*+1/2, −*z*+1/2.
